# Identification of Key Modules and Hub Genes Involved in Regulating the Color of Chicken Breast Meat Using WGCNA

**DOI:** 10.3390/ani13142356

**Published:** 2023-07-19

**Authors:** Xing Guo, Hong Zhang, Hao Wang, Xin-Xin He, Jiang-Xian Wang, Wei Wei, Meng Liu, Jin-Mei Xu, Ya-Nan Liu, Run-Shen Jiang

**Affiliations:** College of Animal Science and Technology, Anhui Agricultural University, Hefei 230036, China; guoxing0405@126.com (X.G.);

**Keywords:** meat color, chicken, WGCNA, gene network, hub genes

## Abstract

**Simple Summary:**

To explore the gene networks and key genes involved in the regulation in meat color of chicken breast muscle, weighted gene co-expression network analysis (WGCNA) was performed based on transcriptome data from the pectoralis major muscle of two yellow feather chicken breeds. The results suggest that genes involved in the regulation of mitochondrial activity and lipid oxidation may play a crucial role in the formation of meat color. The present study advanced our understanding of the molecular mechanisms contributing to meat color and identified potential molecular markers for breeding chicken breast meat color.

**Abstract:**

Meat color is one of the most important economic traits in chickens. However, the gene network and regulatory mechanisms contributing to meat color traits in chickens remain largely unknown. In the present study, we performed weighted gene co-expression network analysis (WGCNA) based on RNA-Seq datasets of 16 pectoralis major muscle samples from two yellow-feather chicken breeds to identify the modules and hub genes related to meat color in chickens. A total of 18,821 genes were used to construct the weighted gene co-expression network, and 29 co-expression gene modules were identified. Among these modules, five modules including blue, brown, steel blue, paleturquoise and orange modules were found to be significantly correlated with meat color traits. Furthermore, several genes within the association module involved in the regulation of mitochondrial activity (e.g., ATP5L, UQCR10 and COX7C) and lipid oxidation (e.g., CAV3, RBP4A and APOH) were identified as hub genes that may play a crucial role in the regulation of meat color. These results provide valuable information to improve our understanding of gene expression and regulation in relation to meat color traits and contribute to future molecular breeding for improving meat color in chickens.

## 1. Introduction

Chicken is the primary source of protein for humans in most parts of the world [1]. Due to its low price, low fat and cholesterol content, chicken meat has become the largest source of meat protein consumed, accounting for approximately 35.3% of global meat consumption [2]. To meet the continued increase in demand for chicken meat, the poultry industry made dramatic improvements in meat production through intensive genetic selection for growth rate and meat yield [3]. For example, fast-growing commercial broilers could reach a market weight of 2.5 kg within ~40 days [4], and the deboned breast and leg meat could reach up to 47% of live weight [2]. However, these advances have often been accompanied by a decrease in the meat quality of birds [5,6]. Meat quality is one of the most important economic traits of chicken. With rapid economic growth and improvements in standards of living, meat quality is becoming increasingly important in the final selection of the consumer [7]. However, meat quality is difficult to define because it is a complex concept determined by consumers’ past experiences and cultural backgrounds [8]. Generally, meat quality parameters can be divided into four major categories: sensory quality parameters (e.g., meat color, amount and distribution of fat, texture of the meat), technological quality parameters (e.g., pH, water-holding capacity, protein ratios), eating quality parameters (e.g., tenderness, flavor, juiciness), and reliance quality parameters (e.g., safety, nutrition, price, animal welfare) [8,9]. Among these parameters, meat color is one of the most important factors influencing purchase decisions [10], as consumers use meat color as an indicator of freshness, wholesomeness, and eating quality [11]. Myoglobin (Mb) is the primary sarcoplasmic heme protein responsible for the meat color, whereas hemoglobin and cytochrome also contribute to meat color, but only to a lesser extent [10]. Thus, the discoloration of meat mainly depends on the concentration and redox state of myoglobin in the meat [12].

Chicken muscle is unique because it exhibits dramatic extremes in color, with breast meat displaying a pale pink color, while thigh and leg meat appear dark red [11]. This is because the leg and thigh meats have a high proportion of oxidative fibers, which contain more myoglobin, while the breast meat is composed almost entirely of fast glycolytic fibers [13]. Many factors were shown to influence color of poultry meat, such as bird sex, age, genetics, feed, handling, stress, slaughter, chilling and processing procedures, chemical exposure, cooking temperature, additives, pH, irradiation and freezing [7,11,13]. Among these, genetics is the dominant factor affecting the color of poultry meat. Heritability estimates showed that the heritability values of lightness (L*), redness (a*) and yellowness (b*) ranged from 0.25 to 0.35 [14], suggesting that genetic selection should be useful to improve chicken meat color. Recently, studies focused on identifying genes and quantitative trait loci associated with meat color traits [15,16,17]. For example, genome-wide association studies (GWAS) analysis based on chicken 60 K SNP chip data revealed that several candidate genes are associated with meat color in F2 populations derived from Beijing–You chickens and Cobb–Vantress, such as collagen, type I, alpha 2 (COL1A2), proteasome 26S subunit, non-ATPase, 12 (PSMD12) and karyopherin subunit alpha 2 (KPNA2), are related to lightness, whereas FtsJ homolog 3 (FTSJ3) is associated with yellowness [16]. Combined with selection signature and differentially expressed gene analyses, 16 candidate genes involved in the regulation of chicken breast meat color were identified [15]. As a quantitative trait, meat color is affected by polygenic and are involves many regulators. Despite many efforts, our understanding of the regulatory network contributes to meat color traits remains largely unknown.

In China, chicken meat is mainly obtained from commercial white-feather broilers and indigenous yellow-feather chickens [18]. Due to their distinctive flavor, yellow-feathered chickens are preferred by consumers [19]. Traditionally, yellow-feather chickens were sold as whole live forms. Recently, to interrupt the spread of avian influenza, live poultry markets have been closed in many cities in China and meat color has become more important for the sale of yellow-feather chickens. However, the specific molecular processes involved in the meat color traits of yellow-feather chickens remain obscure. With the rapid development of high-throughput technologies and significant reductions in sequencing costs, weighted gene co-expression network analysis (WGCNA) is becoming a powerful approach to uncovering the genetic basis underlying specific traits. By applying this approach, many co-expressed gene modules with hub genes associated with economically important traits have been identified in chickens [20,21,22].

There are more than one hundred indigenous chicken breeds in China, which is crucial for diversity of biological genetic resources of the world [18]. Among these local breeds, the Huainan and Wannan chickens are both excellent native chicken breeds in Anhui Province of China. Compared with Huainan chickens (cyan beak and cyan shank), the Wannan chickens exhibit yellow beak and yellow shank [23,24]. Both Huainan and Wannan chickens are dual-purpose breeds characteristics in yellow feather, high meat quality, high nutritional value and resistance to rough feeding, and they are extensively raised in central and south of China [23,24,25]. In the present study, we evaluated the gene expression profile and measured the meat color traits of the pectoralis major of two yellow-feather chicken breeds and employed WGCNA to investigate co-expression patterns and hub genes associated with the meat color. Our results will provide further insights into the molecular basis underlying meat color traits and contribute to future genetic breeding strategies to improve meat color.

## 2. Materials and Methods

### 2.1. Experimental Birds and Sample Preparation

A total of 150 one-day old male Huainan and Wannan chickens, respectively, were obtained from the Jianlang poultry breeding Co., Ltd., Hefei, Anhui Province, China. All chickens were reared under a floor litter-rearing system. Feed and water were provided ad libitum throughout the experimental period. All birds were fed a commercial starter diet containing 18.0% CP with 11.50 MJ/kg of ME from 0 to 21 d of age, a grower diet containing 15.0% CP with 10.80 MJ/kg of ME from 22–49 d of age, and a finisher mash containing 14.0% CP with 10.40 MJ/kg of ME from 50 to 112 d of age. At 112 d of age, 8 birds of similar BW from each breed were selected and euthanized by electrical stunning followed by exsanguination. After euthanasia, left pectoralis major muscle samples were collected parallel to the muscle fiber. After removing the external fat and connective tissues, the samples were frozen in liquid nitrogen. Afterward, the breast muscles were all removed manually and weighed.

### 2.2. Meat Color Parameters Determination

Meat color measurements were performed on the left pectoralis major by using an ADCI-WSI chromameter (Chen Tai Ke, Beijing, China) and calibrated with a white and black tile calibration plate. The values of L*, a* and b* for each meat sample were measured in triplicate, and the final value for color evaluation was the average of the three readings.

### 2.3. cDNA Library Construction and RNA Sequencing

Total RNA was extracted from 16 pectoralis major muscle samples using TRIzol reagent (Invitrogen, Carlsbad, CA, USA) following the manufacturer’s protocol. The concentration and integrity of the RNA were measured by electrophoresis and using a NanoDrop spectrophotometer 2000 (Termo Scientific, Wilmington, DE, USA). A total of 16 RNA libraries were constructed with the NEBNext^®^ UltraTM Directional RNA Library Prep Kit for Illumina^®^ (NEB, Ipswich, MA, USA), according to the manufacturer’s instructions. Sequencing was performed using an Illumina NovaSeq 6000 platform with 150-bp paired-end reads.

### 2.4. RNA-Seq Data Analysis

Read quality control was performed using the Btrim [26] with the parameters ‘-s -a 20 -q’. Clean reads were then mapped to the chicken reference genome (GRCg6a) using HISAT2 [27] with default parameters. The bam files were sorted and indexed using Samtools [28]. Reads for each gene were counted using the Python script htseq-count [29]. Gene expression level normalization was performed by ‘varianceStabilizingTransformation’ function of DESeq2 [30].

### 2.5. Weighted Gene Co-Expression Network Analysis

Weighted gene co-expression network analysis was performed using the WGCNA package (v1.7.1) [31] in R software. Briefly, we constructed a weighted co-expression network using the thresholding power β (β = 1 to 20) to find an optimal soft-thresholding power to transform the co-expression similarity into adjacency. After analysis, the best power was set to 7 to balance the scale-free property of the co-expression network. The cutreeDynamamic function with the parameters “minModuleSize = 30, deepSplit = 2, pamRespectsDendro = F” was used for module detection, and cutHeight = 0.3 was used to merge modules. Phenotype data related to meat quality measurements and gene modules were quantified using Pearson correlation and significant consensus modules were identified with the correlation coefficient |R| ≥ 0.51 and *p* < 0.05. The signedKME function was used to calculate the kME (eigengene connectivity) for each gene. The top 30 connects ranked by kME values of each module were identified as hub genes and visualized using Cytoscape (v3.9.1) [32]. The expression levels of top 30 hub genes in core modules were quantified using the TPM (transcripts per million) values [33].

### 2.6. Functional Enrichment Analysis

Kyoto Encyclopedia of Genes and Genomes (KEGG) and Gene Ontology (GO) enrichment analysis of the hub genes was performed using an online annotation tool g: Profiler [34]. The GO terms or KEGG pathways with a Benjamini–Hochberg FDR *p*-value of 0.05 was considered as the significance threshold to identify the functional categories.

### 2.7. Quantitative RT-PCR Analysis

To verify whether the hub genes were associated with the meat color traits, 8 pectoralis major muscle samples from 817 broilers (sampled at market age of 50 d and meat color were measured), together with 8 samples from Wannan and Huainan chickens selected for RNA-seq analysis, respectively, were used for qPCR analysis. For each sample, 1 µg of total RNA from the pectoralis major muscles for each sample was used to generate cDNA using a cDNA synthesis kit (Vazyme, Nanjing, China) according to the manufacturer’s instructions. Six genes were selected to validate the mRNA expression levels among different breeds. The specific primers for gene amplification are listed in Table S1. The qPCR analysis was performed on an ABI Prism 7500 instrument (Applied Biosystems, Carlsbad, CA, USA) using SYBR Green Supermix (Vazyme, Nanjing, China). Relative gene expression levels were determined by the 2^−ΔΔCt^ method [35] using the GAPDH gene for normalization.

### 2.8. Statistical Analysis

Statistical significance of meat color traits between Wannan and Huainan chickens was tested by performing *t*-tests using SPSS26 for Windows statistical software package (SPSS Inc., Chicago, IL, USA). One-way ANOVA and the post hoc Duncan multiple range test were used to compare the qPCR quantitative expression data and the meat color trait data among the 817 broilers, Wannan and Huainan chickens. Data are expressed as Mean ± SD. The threshold for significance was set at *p* < 0.05.

## 3. Results

### 3.1. The Meat Color of Two Breeds

The meat color values of L*, a* and b* of Wannan and Huainan chickens are shown in Table 1. Compared with the Huainan chickens, the pectoralis major muscle of Wannan chickens had slightly higher L* values, but the result was not statistically significant (Table 1). There were also no significant differences (*p* > 0.05) in a* and b* values between the two groups (Table 1).

### 3.2. Summary of Transcriptome Data

A total of 16 libraries were sequenced, and a total of 371.41 M clean reads were obtained. The percentages of Q20 bases and Q30 bases were above 97.08% and 92.75%, respectively. The GC content of the 16 samples ranged from 48.26% to 54.65%. After mapping to the chicken reference genome (GRCg6a), the mapped read ratio statistics ranged from 80.1% to 90.4% (Table S2).

### 3.3. Weighted Gene Co-Expression Network Construction and Modules Detection

**Figure 1:**
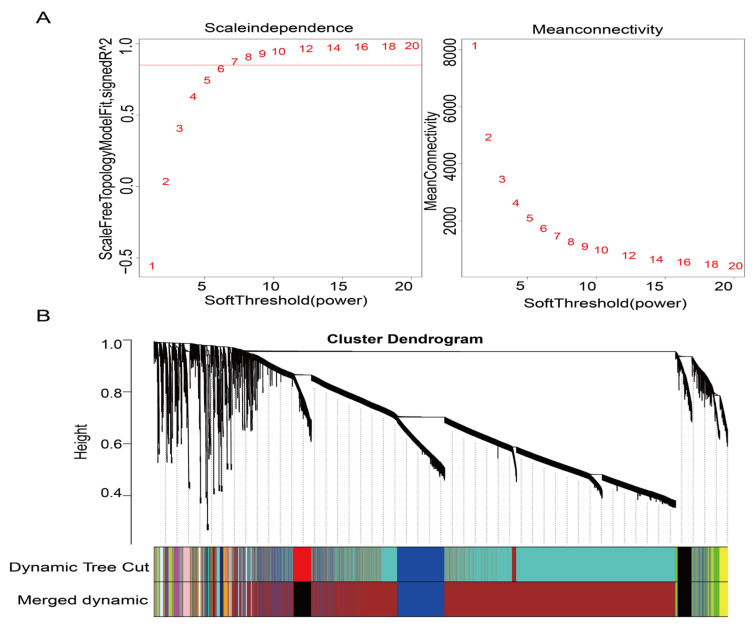
Weighted gene co-expression network analysis of the gene expression dataset. (**A**) Scale-free topology model fit, and gene mean connectivity under different soft threshold powers. The fit index curve indicates that soft threshold power above 7 meets scale−free topology above 0.85. (**B**) Clustering dendrogram of genes and module division by WGCNA. 29 modules represented by colors in the horizontal bar were found using 0.30 threshold merging.

To explore the modules involved in the regulation of meat color traits, a total of 18,821 genes were obtained to build the weighted gene co-expression network. After determining the scale-free topological model and mean connectivity, a soft threshold of seven was considered as the best soft threshold, as the R^2^ of the scale-free network was greater than 0.85 (Figure 1A). After dynamic tree trimming, twenty-nine co-expression gene modules were identified (Figure 1B).

### 3.4. Identification of Meta-Modules Associated with Meat Color Traits

**Figure 2:**
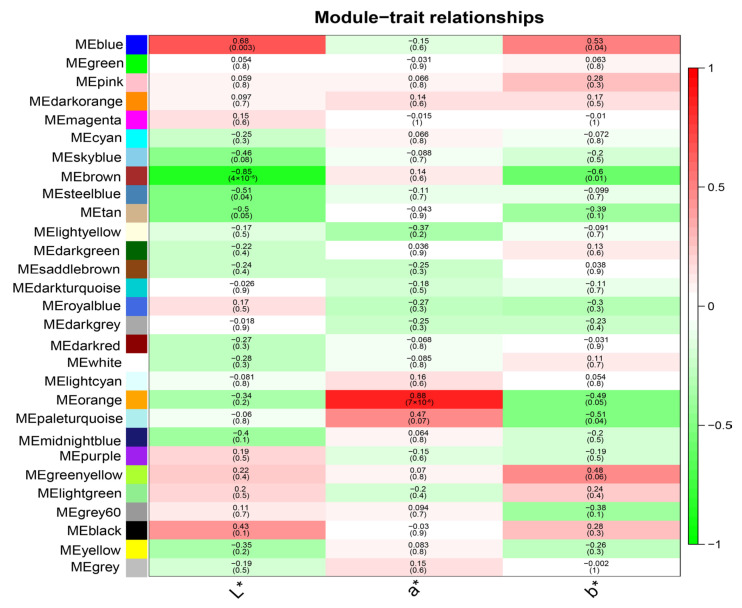
Heat map of the correlation between modules and meat color. Each column represents a trait, and each row denotes an eigengene for a certain module. The matching correlation and *p* value are included in each cell.

To identify co-expression modules associated with meat color parameters, we evaluated the relationship between L*, a* and b* values and the module eigengene (ME). In total, five modules were selected based on |R| ≥ 0.51 and *p* < 0.05. Among these modules, the blue module involving 2725 genes was significantly positively correlated with L* (R = 0.68, *p* = 0.003) and b* (R = 0.53, *p* = 0.04) (Figure 2 and Table S3). A group of 11,874 genes within the brown module negatively correlated with L* (R = −0.85, *p* = 4 × 10^−5^) and b* (R = 0.60, *p* = 0.01) (Figure 2 and Table S3). The steelblue and paleturquoise modules were significantly negatively correlated with L* (R = −0.51, *p* = 0.04) and b* (R = −0.51, *p* = 0.04), respectively. We also observed that the orange module was significantly positively correlated with a* (R = 0.88, *p* = 7 × 10^−6^) (Figure 2).

### 3.5. Functional Enrichment Analysis of Genes in Relevant Modules

**Figure 3:**
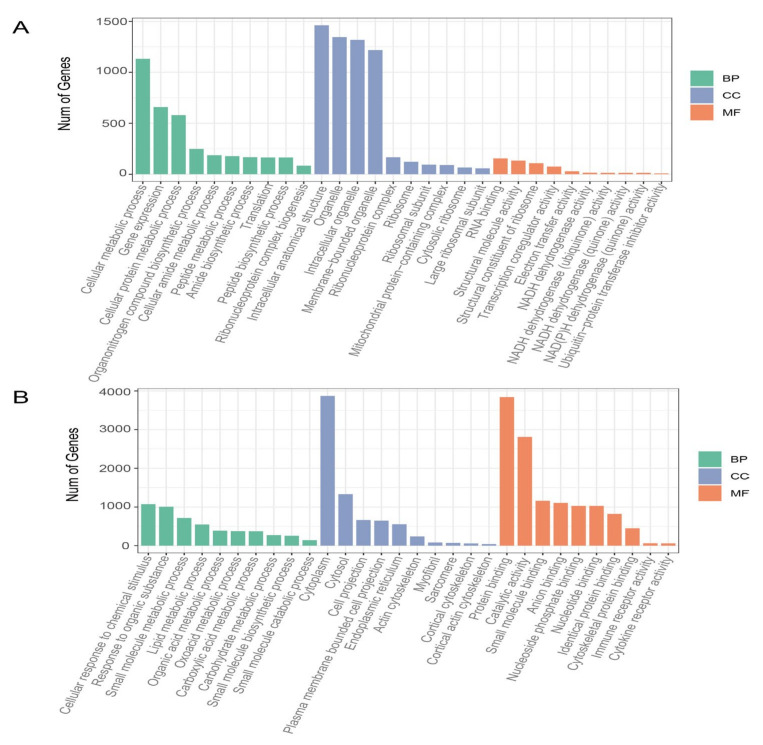
The functional enrichment analysis of genes within the blue (**A**) and brown (**B**) modules. The top 10 significantly enriched molecular function (MF), cellular component (CC) and biological process (BP) of GO terms were presented.

Enrichment analysis was performed for genes in the blue, brown, steelblue, orange and paleturquoise modules. Genes in the blue module were significantly enriched in 221 GO categories and four KEGG pathways, such as “Mitochondrial respiratory chain complex assembly”, “NADH dehydrogenase complex assembly”, “Mitochondrion organization” and “Oxidative phosphorylation”. There were 200 GO categories and 16 KEGG pathways, including “Lipid metabolic process”, “Fatty acid metabolic process”, “Regulation of lipid metabolic process”, and “Peroxisome”, which were significantly enriched in the brown module. The top 10 significantly enriched molecular function (MF), cellular component (CC) and biological process (BP), respectively, of GO terms are illustrated in Figure 3. Detailed results of GO enrichment analyses are presented in Tables S4 and S5. However, no over-represented categories were identified among genes grouped in steelblue, orange and paleturquoise modules.

### 3.6. Identification and Visualization of Hub Genes Related to Meat Color

**Figure 4:**
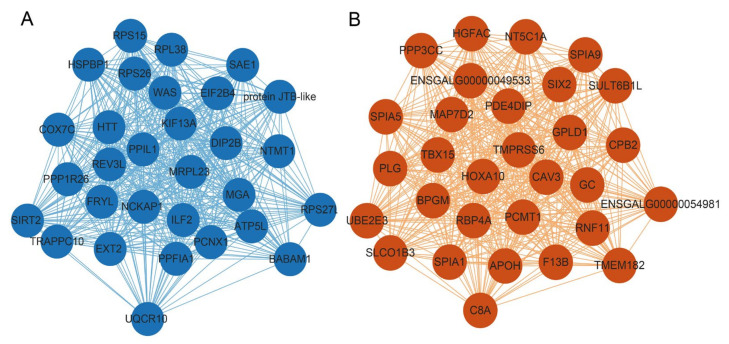
Gene interaction network diagram of the top 30 genes ranked by kME values in the blue module (**A**) and brown module (**B**).

Hub genes can act as the representatives of a module, as indicated by their high eigengene connectivity values. In the present study, the top 30 genes ranked by kME values were identified as hub genes for each module. Cytoscape software (v3.9.1) was used to generate the interaction network diagrams for these hub genes. Among the five modules, we focused on the blue, brown and orange modules that exhibited the highest correlation with L*, a* and b* values. In the blue module, we found that several hub genes, including ATP synthase, H+ transporting, mitochondrial Fo complex subunit G (ATP5L), ubiquinol-cytochrome c reductase, complex III subunit X (UQCR10) and Cytochrome c oxidase subunit 7C (COX7C), were involved in oxidative phosphorylation or ATP metabolism (Figure 4A and Table S6). A few genes involved in lipid metabolism, such as caveolin 3 (CAV3), retinol binding protein 4 A, plasma (RBP4A) and apolipoprotein H (APOH), were identified in the brown module (Figure 4A and Table S6). However, most of the hub genes in the orange module were identified as novel genes, and only a few genes such as aquaporin-8 (AQP8), purinergic receptor P2X3 (P2RX3), transmembrane protein 52B (TMEM52B), interleukin 17C (IL17C) and chymotrypsinogen B2 (CTRB2) were annotated (Figure S1).

### 3.7. Expression Levels of the Top 30 Hub Genes in Core Modules

**Figure 5:**
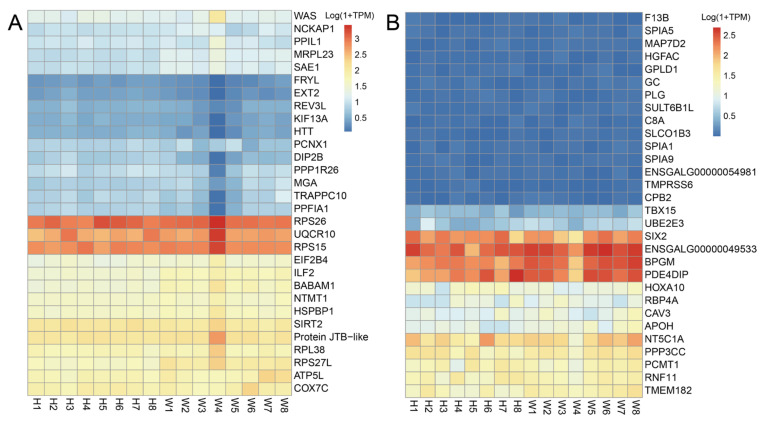
Expression of the top 30 hub genes in the pectoralis major muscle of Huainan (H1–H8) and Wannan (W1–W8) chickens in the blue module (**A**) and brown module (**B**).

We further quantified the expression levels of the top 30 hub genes in the blue, brown and orange modules using the TPM values. A dozen genes in the blue module, including ATP5L, UQCR10, COX7C and ribosomal protein S15 (RPS15) were abundantly expressed in the pectoral muscle of two breeds (Figure 5A). In the brown module, several genes including bisphosphoglycerate mutase (BPGM), SIX homeobox 2 (SIX2) and phosphodiesterase 4D interacting protein (PDE4DIP) were highly expressed, whereas the mRNA abundance of CAV3, RBP4A, APOH and 5′-nucleotidase, cytosolic IA (NT5C1A) were low to moderate levels in the pectoral muscle of both Wannan and Huainan chickens (Figure 5B). However, all the top 30 hub genes of the orange module were expressed at low levels in the pectoral muscle of both Wannan and Huainan chickens (Figure S2).

### 3.8. Verification the Expression Levels of Hub Genes in Different Breeds

**Figure 6:**
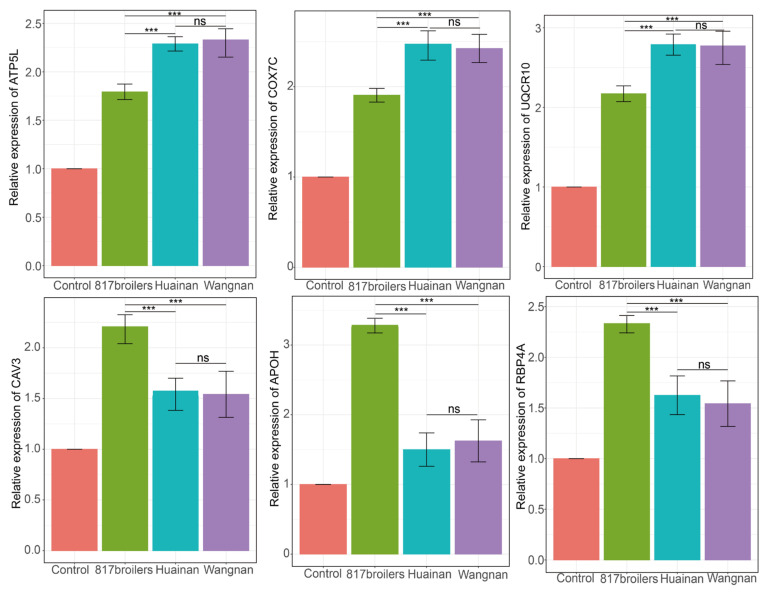
Relative expression levels of the selected six hub genes in the pectoralis major muscle of three breeds. Data analysis was performed using one-way ANOVA, with Duncan multiple range test. ns (no significant), *** (*p* < 0.001).

Three hub genes in the blue module (ATP5L, UQCR10 and COX7C) and three genes in the brown module (CAV3, RBP4A and APOH) were selected to verify the expression levels in 817 broilers, Wannan and Huainan chickens. Firstly, we compared the L*, a* and b* values among the three breeds and the results showed that the 817 broilers had significant lower L* and b* values (*p* < 0.01) when compared with both Wannan and Huainan chickens (Table S7). Secondly, we performed qPCR to quantify the relative expression levels of the six hub genes in the three breeds. The mRNA levels of ATP5L, UQCR10 and COX7C genes were significantly higher in the pectoralis major muscle of both Wannan and Huainan chickens than that of 817 broilers (Figure 6). However, the expression levels of CAV3, RBP4A and APOH were significantly up-regulated in the 817 broilers when compared with the other two breeds (Figure 6). Moreover, the expression levels of ATP5L, UQCR10 and COX7C were significantly positively correlated with L* and b* values (Table S8), whereas the expression levels of CAV3, RBP4A and APOH were significantly negatively correlated with L* and b* values (Table S9).

## 4. Discussion

Improving meat quality has always been the broiler industry’s goal. Among the meat quality parameters, the appearance quality properties ultimately influence the consumer’s purchase [9]. Meat color is considered to be the most important appearance quality attribute and can be measured using a colorimeter that measures the Commission International d’Eclairage (CIE) L*, a* and b* values [13]. L*, a* and b* values of the meat are mainly affected by myoglobin concentration and the chemical state of the myoglobin [36,37] and mitochondrial activity is considered to be one of the most prominent factors to affecting CIE values [38]. In the present study, we found that the blue module was significantly positively correlated with L* and b* values, respectively. We further used kME values to screen the top 30 hub genes in blue module. Among these hub genes, three genes (ATP5L, UQCR10 and COX7C) were highly expressed in the pectoral muscle of both Wannan and Huainan breeds and were significantly enriched in the categories of “Oxidative phosphorylation”, “ATP metabolic process” and “ATP biosynthetic process”. We also performed qPCR analysis using the three breeds to verify whether the expression levels of the three genes were associated with the L* and b* values of breast meat. The results showed that the chickens with higher L* and b* values had a higher expression level of the three genes. Furthermore, correlation analysis suggested that the expression levels of ATP5L, UQCR10 and COX7C were significantly positively correlated with L* and b*values. These results suggest that ATP5L, UQCR10 and COX7C may contribute to the L* and b* values of meat color. ATP5L encodes the gamma subunit of mitochondrial ATP synthase, which is involved in oxidative phosphorylation [39], and decreased expression of ATP5L resulted in dysfunction in ATP metabolism in rats [40]. UQCR10, a functional protein in mitochondrial complex III [41], is a member of the multi-subunit phanquinone-cytochrome c reductase complex [42] and is crucial for respiratory electron transport [43], which is used by ATP synthase during oxidative phosphorylation to produce the majority of the cellular ATP [44]. COX7C is a member of the cytochrome c oxidase complex, which plays a critical role in maintaining mitochondrial membrane potential and promoting ATP generation during oxidative phosphorylation [45]. Knockdown of COX7C reduced the mitochondrial membrane potential, whereas upregulation of COX7C promoted ATP synthesis and improved mitochondrial respiratory capacity [45]. The identification of these hub genes suggests that affecting mitochondrial activity may be an important way in which the blue module contributes to L* and b* values.

Lipid oxidation was demonstrated to be another dominant factor affecting meat color [10]. Many studies demonstrated the interaction between lipid oxidation and myoglobin oxidation [38]. The process of lipid oxidation was reported to increase meat discoloration, and the underlying mechanisms were mainly due to the reaction of primary and secondary products derived from lipid oxidation [38]. Therefore, inhibition of lipid oxidation could improve color stability [38]. The brown module was found to be significantly negatively correlated with L* and b*. Among the hub genes of the brown module, several genes, including CAV3, RBP4A and APOH, were overrepresented in “Lipid metabolic process”, and “Cellular lipid metabolic process”. qPCR analysis found that the 817 broilers with higher L* and b* values had a higher expression level of the CAV3, RBP4A and APOH genes. Further analysis showed that the expression levels of CAV3, RBP4A and APOH were significantly negatively correlated with L* and b* values. These results suggest that ATP5L, UQCR10 and COX7C may negatively regulate L* and b* values of meat color. APOH is a plasma glycoprotein that exists both as a free protein and is associated with lipoproteins [46]. APOH is known to bind to specific lipoproteins and activate lipoprotein lipase, which is closely associated with lipid metabolism [47]. In duck myoblasts, APOH regulates lipid deposition by inhibiting fatty acid beta-oxidation and promoting fatty acid biosynthesis [48]. CAV3 is a member of the caveolin gene family, expressed mainly in skeletal and smooth muscle cells as well as cardiac myocytes [49]. CAV3 regulates free fatty acid (FA) uptake by interacting with the FA transport protein CD36 (CD36 molecule) [50]. CD36 is a multifunctional receptor that mediates the uptake of long-chain fatty acids (LCFAs) and oxidized lipids [51]. The transport and oxidation of LCFAs in skeletal muscle is upregulated by the expression of CD36 [52]. RBP4A, also known as RBP4, functions as a transporter of vitamin A in the blood, from the liver and adipose tissue to the peripheral tissues [53]. Overexpression of RBP4 in mice impairs mitochondrial fatty acid oxidation by significantly decreasing the mRNA levels of several genes, including carnitine palmitoyl transferase 1A (CPT1A), peroxisome proliferator activated receptor alpha (PPARA) and β-hydroxyacyl coenzyme a dehydrogenase (β-HAD), which are involved in mitochondrial β-oxidation [54].

The orange module was found to be significantly negatively correlated with a* values. However, all the top 30 hub genes of the orange module are expressed at low levels in the pectoral muscle of both yellow-feather chickens. Moreover, most of the hub genes in the orange module were identified as novel genes, and only a few genes were annotated. For example, AQP8, a member of the water channel trans-membrane proteins family, was found to be expressed in the inner mitochondrial membrane and is essential for mitochondrial structure [55]. AQP8 plays an important role in maintaining normal mitochondrial function, and knockdown of mtAQP8 expression impaired H_2_O_2_ mitochondrial release and increased mitochondrial ROS [55]. The release of mitochondrial ROS was an important factor for muscle oxidative stress, and the decrease in muscle antioxidant capacity [56]. However, antioxidants can protect myoglobin for against oxidation [57,58]. P2RX3 is a member of the P2X ionotropic receptor family and is activated by ATP [59]. However, the mechanism by which P2RX3 contributes to meat color remains unclear, and one potential mechanism is that P2X receptors have an important role in signal transduction in O2-sensing [58].

## 5. Conclusions

In summary, in this study, 16 RNA libraries were constructed and sequenced from the pectoralis major muscles of two yellow-feather chicken breeds. Based on the transcriptome data, WGCNA analysis was used to identify the co-expression patterns and hub genes associated with the meat color. In total, five modules were identified as associated with meat color traits. Particularly, several hub genes that are involved in the regulation of mitochondrial activity (e.g., ATP5L, UQCR10 and COX7C) and lipid oxidation (e.g., CAV3, RBP4A, and APOH) were identified as potential regulators of meat color. Overall, our study provides a useful gene expression data resource for the chicken pectoralis major and contributes to future genetic breeding strategies to improve the meat color of yellow feather chickens.

## Figures and Tables

**Table 1:** Comparison of meat color traits between Wannan and Huainan chickens ^1^.

Item	Wannan	Huainan	*p*-Value
L*	54.60 ± 1.92	52.20 ± 2.63	0.058
a*	8.84 ± 2.54	9.66 ± 2.27	0.508
b*	15.89 ± 3.92	15.70 ± 2.83	0.914

^1^ Data are presented as the Mean ± SD.

## Data Availability

The RNA-seq data from this study were deposited in the GSA (http://gsa.big.ac.cn/) with accession no. CRA011027 (accessed on 13 May 2023).

## Supplementary Materials

The following supporting information can be downloaded at: https://www.mdpi.com/article/10.3390/ani13142356/s1. The data presented in this study are contained within the article or Supplementary Materials. Figure S1: Gene interaction network diagram of the top 30 kME values value in the orange module; Figure S2: Expression of the top 30 hub genes in the pectoralis major muscle of Huainan (H1–H8) and Wannan (W1–W8) chickens in the orange module; Table S1: qPCR primers for the six hub genes; Table S2: Summary of RNA-seq data; Table S3: Statistical table of module gene numbers; Table S4: Functional enrichment analysis of the protein-coding genes in the blue module; Table S5: Functional enrichment analysis of the protein-coding genes in the brown module; Table S6: The KME value of each gene in the 29 modules; Table S7: Comparison of meat color traits among different breeds; Table S8: The Pearson correlation between meat color (L* and b* values) and the expression levels of three hub genes in the blue module; Table S9: The Pearson correlation between meat color (L* and b* values) and the expression levels of three hub genes in the brown module.
