# Supplementary material for: Identification of Key Modules and Hub Genes Involved in Regulating the Color of Chicken Breast Meat Using WGCNA
Source: Animals (Basel). 2023 Jul 19;13(14):2356. doi: 10.3390/ani13142356 (PMC10376702; doi:10.3390/ani13142356)
Supplement: Supplementary file 1 [file animals-13-02356-s001.zip › Supplementary-Figure.pdf]

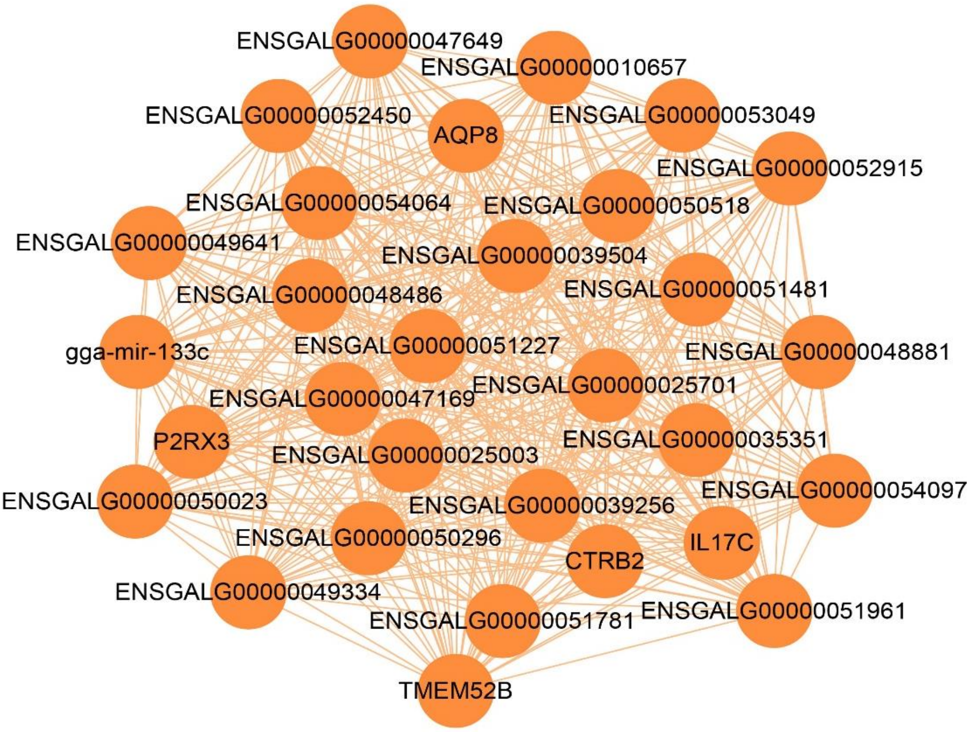

Figure S1. Gene interaction network diagram of the top 30 kME values value in the orange module.

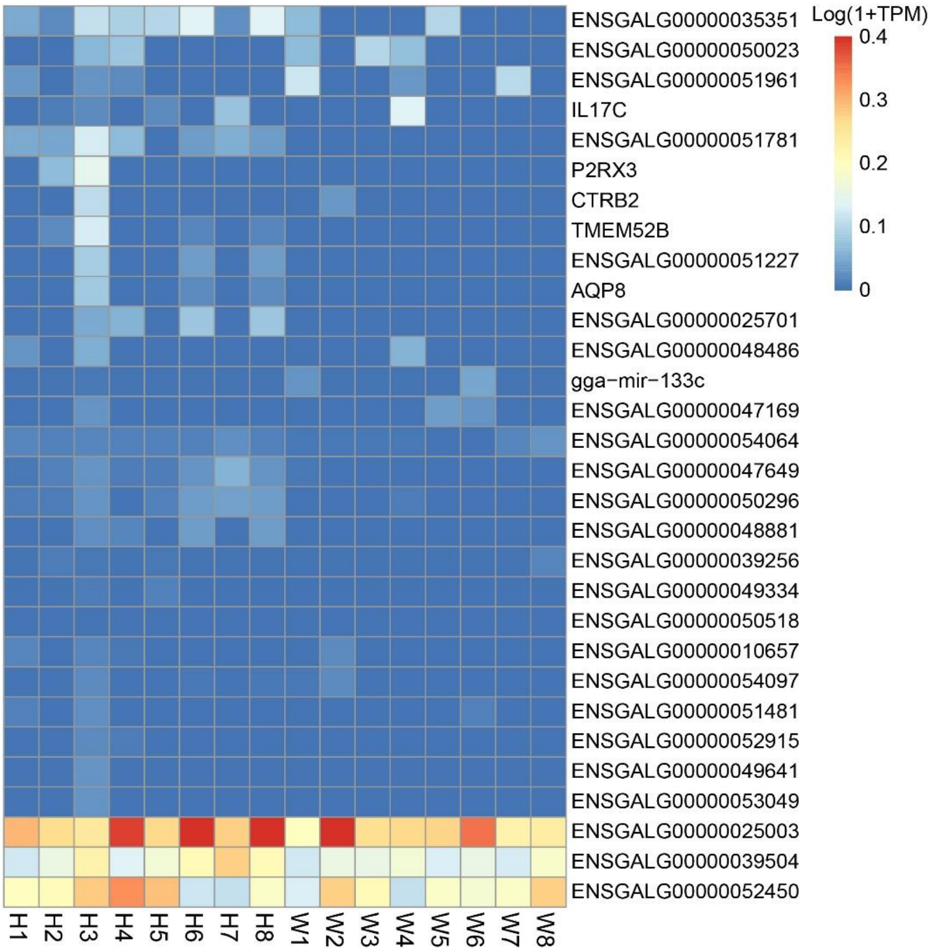

Figure S2. Expression of the top 30 hub genes in the pectoralis major muscle of Huainan (H1-H8) and Wannan (W1-W8) chickens in the orange module.
